# Association between Type 2 Diabetes Loci and Measures of Fatness

**DOI:** 10.1371/journal.pone.0008541

**Published:** 2010-01-01

**Authors:** Slavica Pecioska, M. Carola Zillikens, Peter Henneman, Pieter J. Snijders, Ben A. Oostra, Cornelia M. van Duijn, Yurii S. Aulchenko

**Affiliations:** 1 Department of Epidemiology and Biostatistics and Clinical Genetics, Erasmus Medical Center, Rotterdam, The Netherlands; 2 Department of Internal Medicine, Erasmus Medical Center, Rotterdam, The Netherlands; 3 Department of Human Genetics, Leiden University Medical Centre, Leiden, The Netherlands; 4 Institute of Cytology and Genetics, SD RAS, Novosibirsk, Russia; Peninsula Medical School, United Kingdom

## Abstract

**Background:**

Type 2 diabetes (T2D) is a metabolic disorder characterized by disturbances of carbohydrate, fat and protein metabolism and insulin resistance. The majority of T2D patients are obese and obesity by itself may be a cause of insulin resistance. Our aim was to evaluate whether the recently identified T2D risk alleles are associated with human measures of fatness as characterized with Dual Energy X-ray Absorptiometry (DEXA).

**Methodology/Principal Findings:**

Genotypes and phenotypes of approximately 3,000 participants from cross-sectional ERF study were analyzed. Nine single nucleotide polymorphisms (SNPs) in *CDKN2AB*, *CDKAL1*, *FTO*, *HHEX*, *IGF2BP2*, *KCNJ11*, *PPARG*, *SLC30A8* and *TCF7L2* were genotyped. We used linear regression to study association between individual SNPs and the combined allelic risk score with body mass index (BMI), fat mass index (FMI), fat percentage (FAT), waist circumference (WC) and waist to hip ratio (WHR). Significant association was observed between rs8050136 (*FTO*) and BMI (*p* = 0.003), FMI (*p* = 0.007) and WC (*p* = 0.03); fat percentage was borderline significant (*p* = 0.053). No other SNPs alone or combined in a risk score demonstrated significant association to the measures of fatness.

**Conclusions/Significance:**

From the recently identified T2D risk variants only the risk variant of the *FTO* gene (rs8050136) showed statistically significant association with BMI, FMI, and WC.

## Introduction

Type 2 diabetes mellitus (T2D) is a metabolic disorder characterized by disturbances of carbohydrate, fat and protein metabolism and insulin resistance [1]. The effects of T2D include long-term damage, dysfunction and failure of various organs. The global prevalence of diabetes is currently estimated as 2.8% (171 millions patients worldwide), and this figure projects to at least 4.4% (366 millions) at year 2030 [2].

The majority of T2D patients are obese and obesity by itself may be a cause of insulin resistance [3]. The rapid increase in prevalence of T2D is thought to be at least partly explained by the change in environmental factors (nutrition and life style) acting on genetically susceptible individuals, who may become obese and consequently develop insulin resistance and T2D. This hypothesis received support from the fact that several candidate genes known for T2D, such as *PPARG* [4] and *LPIN2* [5], are involved in the development of fat tissue, while *FTO* [6] gene may be involved in regulation of food intake.

Up until recently only few genes were unequivocally implicated in risk of Type 2 Diabetes. In the last year, large progress has been made through the genome-wide association studies. Besides confirming the previously known T2D loci *PPARG* [4], *TCF7L2* [7] and *KCNJ11* [8], the GWA studies have identified new loci *CDKAL1*, *CDKN2A/CDKN2B*, *IGF2BP2*, *FTO*, *SLC30A8* and *HHEX* [9]–[12] that influence the risk of T2D. A recently published meta-analysis introduced yet another 6 loci associated with T2D, *JAZF1*, *CDC123-CAMK1D*, *TSPAN8-LGR5*, *THADA*, *ADAMTS9* and *NOTCH2* [13].

We have selected polymorphisms in nine T2D genes, *CDKAL1*, *CDKN2A/CDKN2B*, *FTO*, *HHEX*, *IGF2BP2*, *KCNJ11*, *PPARG*, *SLC30A8* and *TCF7L2*. Two of these genes, *PPARG* and *KCNJ11*, are well-established candidate genes involved in T2D pathways, while the other seven were discovered in initial GWAS and therefore should explain a large fraction of T2D cases.

## Results and Discussion

Association between the polymorphisms and the measures of fatness was studied in ∼3,000 participants from The Erasmus Rucphen Family (ERF) study. The ERF study is a family based study of a recent genetically isolated population located in the South West of the Netherlands [14]. Participants that were classified as diabetic, according to the use of anti-diabetic medication (oral medication or insulin) or had fasting plasma glucose levels ≥7.0 mmol/l, were excluded from the analyses (n = 145). We have studied association of the polymorphisms to fat mass index (FMI) and to fat percentage (FAT), as assessed by Dual Energy X-ray Absorptimetry (DEXA), and to body mass index (BMI), waist circumference (WC) and waist to hip ratio (WHR). In total, 3035 participants had complete genotypic and phenotypic information and were used in analysis. In this study, at nominal *p* = 0.05, we had 97% power to detect influence explaining as little as 0.5% of the trait's variation, 85% power to detect 0.3% of the trait's variation and 41% power to detect 0.01% of the trait's variation.

Characteristics of the population studied are presented in Table 1 and SNPs details are presented in Table 2.

**Table 1:** General characteristics of the population studied.

Trait	Women (N = 1721)	Men (N = 1314)
Age – years	50.33±15.95	49.97±14.93
Height – cm	161.87±6.48	174.99±7.19*
Weight – kg	69.11±13.44	83.19±13.91*
BMI – kg/m^2^	26.38±4.87	27.15±4.13*
FMI – kg/m^2^	10.06±3.68	7.19±2.79*
FAT %	38.64±7.58	26.71±7.13*
WC-cm	81.32±11.87	93.37±11.44*
WHR	0.80±0.08	0.94±0.08*

All values are means or percentages±SD. BMI = body mass index, FMI = fat mass index, FAT = fat percentage, WC = waist circumference, WHR = waist to hip ratio. * = p<0.05.

**Table 2:** T2D susceptible alleles and genotype frequencies for the ERF participants.

Locus	Chr	SNPs	Position (bp)	Alleles	N	Allele Freq. (n)	Genotype Frequencies (n)	Phw	Freq. Ref.
				A1/A2		A1	A2	A1A1	A1A2	A2A2		
IGF2BP2	3	rs4402960	186994389	T/G	2891	28.8	71.2	8.8	40.1	51.1	0.238	0.29–0.30
						(833)	(2058)	(253)	(1159)	(1479)		
CDKN2B	9	rs10811661	22124094	T/C	2888	86.6	13.4	75.1	23	1.9	0.748	0.83–0.85
						(2501)	(387)	(2168)	666	54		
CDKAL1	6	rs7754840	20769229	C/G	2871	32.7	67.3	11.4	43.3	45.3	0.127	0.31–0.36
						(993)	(2115)	(326)	(1229)	(1316)		
PPARG	3	rs1801282	12368125	C/G	2955	90.1	9.9	81.3	17.7	1	0.757	0.82–0.86
						(2662)	(293)	(2402)	(523)	(30)		
SLC30A8	8	rs13266634	118253964	C/T	2934	69.2	30.8	47.4	43.6	9	0.260	0.61–0.75
						(2030)	(904)	(1391)	(1278)	(265)		
HHEX	10	rs1111875	94452862	C/T	2940	59.2	40.8	34.9	48.4	16.7	0.939	0.52–0.64
						(1740)	(1200)	(1028)	(1423)	(489)		
KCNJ11	11	rs5219	17366148	T/C	2935	33.6	66.4	11.4	44.4	44.2	0.804	0.46–0.47
						(986)	(1949)	(335)	(1304)	(1296)		
TCF7L2	10	rs7903146	114748339	T/C	2916	28.5	71.5	7.8	41.3	50.9	0.467	0.18–0.26
						(831)	(2084)	(228)	(1204)	(1482)		
FTO	16	rs8050136	52373776	A/C	2942	43.1	56.9	17.2	51.8	31.0	0.011	0.38
						(1268)	(1674)	(505)	(1524)	(913)		

Chr. = Chromosome, Risk allele for type 2 diabetes underlined. Phw = p value for hardy-weinberg. Freq. Ref. = reference frequency.

In our association study each SNP was individually tested for association to each of the measures of fatness traits. There was significant association between the risk allele of the FTO gene and most of the measures of fatness traits. The significant association was observed between rs8050136 and BMI *p* = 0.003, FMI *p* = 0.007, WC *p* = 0.03 and border line significance for FAT *p* = 0.053. The results of the individual SNP analyses are presented in Table 3.

**Table 3:** Individual effects of T2D polymorphisms.

SNP Locus	Risk Alle.	Freq.	TRAIT														
			BMI			FMI			FAT			WC			WHR		
			β	Se	p	β	se	p	β	se	p	β	se	p	β	se	p
rs13266634	C	0.69	−0.18	0.14	0.28	−0.1	0.1	0.43	−0.09	0.23	0.76	−0.13	0.34	0.68	−1.33E-03	2.07E-03	0.59
SLC30A8																	
rs10811661	T	0.87	−0.04	0.18	0.87	−0.02	0.14	0.92	−0.01	0.3	0.99	0.06	0.46	0.91	2.02E-03	2.78E-03	0.54
CDKN2AB																	
rs7754840	C	0.33	0.15	0.13	0.35	0.08	0.1	0.5	0.17	0.22	0.52	0.2	0.33	0.61	−4.29E-04	2.00E-03	0.86
CDKAL1																	
rs4402960	T	0.29	0.17	0.14	0.29	0.07	0.1	0.56	−0.06	0.22	0.83	0.35	0.33	0.38	8.09E-04	2.05E-03	0.74
IGF2BP2																	
rs1111875	C	0.59	0.11	0.13	0.47	0.06	0.09	0.58	0.16	0.21	0.53	0.14	0.31	0.71	6.92E-04	1.91E-03	0.76
HHEX																	
rs1801282	C	0.9	0.25	0.22	0.31	0.22	0.16	0.24	0.65	0.35	0.13	0.38	0.52	0.53	−8.17E-05	3.17E-03	0.98
PPARG																	
rs5219	T	0.34	−0.04	0.13	0.81	2.1E-03	0.1	0.99	0.13	0.22	0.64	0.16	0.33	0.68	1.67E-03	1.99E-03	0.48
KCNJ11																	
rs7903146	T	0.28	0.1	0.14	0.56	0.08	0.1	0.5	0.28	0.23	0.33	0.39	0.34	0.34	2.32E-03	2.09E-03	0.35
TCF7L2																	
rs8050136	A	0.43	0.45	0.13	3.0E-03*	0.31	0.09	7.0E-03*	0.51	0.21	5.3E-02	0.79	0.32	3.0E-02*	1.02E-03	1.96E-03	0.66
FTO																	

All analyses are adjusted for sex and age. BMI = body mass index, FMI = fat mass index, FAT = fat %, WC = waist circumference, WHR = waist to hip ratio. b = beta coefficient, se = standard error, p = p value, * = p<0.05.

To estimate the combined effect of all risk alleles on the measure of fatness traits, we computed the risk allelic score, estimated as the number of T2D risk alleles in the genotype of an individual. The mean allelic score was 9.44 (SD = 1.88, range: 3–16). Though effects of the score on every fatness trait were positive, there was no evidence for significant association between the risk score and any of the traits. The lowest, marginally nominally significant, *p*-values were observed for the association of the risk allelic score with body mass index (*p* = 0.067) and waist circumstance (*p* = 0.065). Results of the analyses of the risk allelic score are presented in Table 4.

**Table 4:** Effects of the risk allelic score on traits.

Traits	Intercept±se	Sex		Age		Score	
		βs±se	ps	βa±se	pa	β±se	p
BMI	22.25±0.60	0.79±0.20	6.65E-05	0.06±0.01	<2E-16	0.12±0.05	0.067
FMI	7.54±0.44	−2.82±0.15	<2E-16	0.04±0.01	1.15E-12	0.08±0.04	0.086
FAT	33.75±0.98	−11.77±0.32	<2E-16	0.06±0.01	4.71E-08	0.20±0.09	0.065
WC	65.19±1.46	12.26±0.48	<2E-16	0.28±0.02	<2E-16	0.27±0.13	0.081
WHR	0.66±0.01	0.14±2.9E-03	<2E-16	2.9E-03±1.0E-04	<2E-16	8.8E-04±7.7E-04	0.336

**β**s = coefficient for sex, **β**a = coefficient for age, **β** = coefficient for risk allelic score, pi = p-value for intercept, ps = p = value for sex, pa = p-value for age, p = p-value for measure of fatness traits.

We analyzed the data stratified by disease status (type 2 diabetes yes/no) and by sex. However, there was no evidence for statistically significant association in these sub-sets. We also performed an extreme analyzes on our data, assessing an association between the measures of fatness and selected population (10% top vs. 10% bottom levels of the population). Comparing this two extreme groups we did not see escalations from the previous results. The results of the extreme analyzes are presented in Tables S1 and S2.

Our study is among the first to assess association between recently discovered T2D loci and measures of fatness as characterized with Dual Energy X-ray Absorptiometry (DEXA). We found significant association between rs8050136 (*FTO*) and BMI (*p* = 0.003), FMI (*p* = 0.007) and WC (*p* = 0.03); fat percentage was borderline significant (*p* = 0.053). No other SNPs alone or combined in a risk score demonstrated significant association to the measures of fatness.

Absence of association between T2D loci and the measures of fatness may be at least partly explained because some of these were discovered using cohorts of lean subjects. This especially concerns loci discovered in the study of Sladek et al. [9] where discovery stage was based on lean participants with young onset. Later, it was shown that *SLC30A8* exhibited its effect mostly in the lean participants [15]. However, the loci such as *TCF7L2*, *HHEX*, *PPARG*, *FTO*, *KCNJ11*, *CDKN2B*, *IGNF2BP2*, and *CDKAL1*, were discovered in GWAS [10]–[13] where cases were not selected for BMI.

In agreement with other studies [16] we found no association between *PPARG* and the measures of fatness. However, the variant of *PPARG* (rs1801282) shows some controversy in the associations with obesity. Even though the variant has been replicated many times as a risk one for type 2 diabetes and severe insulin resistance [4], its effect on BMI is unclear. Some studies showed that Pro12Ala allele, associated with T2D, is associated with lower body mass index [17], but other studies showed that the same polymorphism is not associated with childhood or adult obesity [16]. Despite this controversy, the biological role of *PPARG* in the fat cell differentiation and lipodistrophy is strongly proven. Cauchi *et al.* [15] recently suggested that the effect of genetic polymorphisms, including *PPARG*, which have a role in adipocyte differentiation, maturation and action, and lead to insulin resistance, might be worsened by the effect of obesity.

In this study we also confirm an association between the *FTO* gene and measures of fatness. Even thou the *FTO* gene was first discovered as T2D gene in an analysis not adjusted for BMI; after adjustment for BMI the effect on T2D was abolished, confirming the effect of *FTO* on BMI [18]. Another gene which may be involved in fat tissue development and T2D is the *LPIN2* gene [5], which is the human homologues of mouse gene *Lpin1* involved also in the human lipodistrophy.

To conclude, though *FTO* locus provides a strong example of T2D locus with an effect on measures of fatness, in general the direct effects of T2D loci on measures of fatness are limited.

## Materials and Methods

### Study Population

Subjects were participants of the Erasmus Ruchpen Family (ERF) study. The Erasmus Ruchpen Family (ERF) study is a family based study of a genetically isolated population located in the South West of the Netherlands. The population was founded in the middle of the 18^th^ century by less than 400 people and experienced exponential growth and minimal immigration during the last decades. Currently, the population consists of more than 20,000 inhabitants scattered across eight adjacent villages. For the ERF study, with the help of genealogical records, twenty couples that had at least 6 children baptized in the community church between 1850–1900 were identified. All living descendants of these couples and their spouses were invited to participate in the study.

The present study is based on ∼3,000 participants. All participants gave an informed written consent, and the Medical Ethical Committee of the Erasmus Medical Center Rotterdam, approved the study protocol.

### Data Collection

All participants of the ERF study were invited for extensive clinical examinations at the research center. Fasting blood samples, anthropometric measurements and personal interviews were obtained with medical practitioners.

For the current study measures of fatness and anthropometric measurements were used. Height and weight were measured with the participants dressed in light underclothing. Fat mass and lean mass were assessed using Dual Energy X-ray Absorptiometry (DEXA). Body mass index (BMI) was calculated as weight divided by height squared (kg/m^2^). Accordingly, FMI was calculated as fat mass divided by height squared (kg/m^2^) and LMI as lean mass divided by height squared.

### Genotyping

We genotyped the following SNPs in all participants: rs7754840 (*CDKAL1*), rs10811661 (*CDKN2AB*), rs8050136 (FTO), rs1111875 (*HHEX*), rs4402960 (*IGF2BP2*), 5215 (*KCNJ11*), rs1801282 (*PPARG*), rs13266634 (*SLC30A8*) and rs7903146 (*TCF7L2*). The genotyping was performed using TaqMan allelic discrimination Assays-By-Design (Applied Biosystems, Foster City, CA, http://store.appliedbiosustems.com). Genotypes were determined in 2-ng genomic DNA. Reactions were performed on the TaqMan Prism 7900HT platform.

### Statistical Analyses

All statistical analyses were performed using statistical package R (www.r-project.org). Prior to analyses a quality control was performed on all SNPs, assessing a minor allele frequency (all>0.05), call rate (all>0.94) and Hardy-Weinberg Equilibrium (Tab. 2), and presence of duplicates. The association analyses were done using GenABEL package [18] for R. SNPs were individually tested for association with BMI, FMI, fat percentage, waist circumference and waist to hip ratio using the linear regression model, adjusted for sex and age. The p-values were corrected by the inflation factor using genomic control method [19]. The results were not corrected for multiple testing. To estimate the combined effect of all risk alleles, the risk allelic score was calculated as total number of risk alleles (alleles associated with increase T2D) present in the genotype of the study participant. The linear regression model, adjusted for sex and age, was applied to estimate the association of the risk allelic score on the five measures of fatness.

## Supporting Information

Table S1 Individual effects of T2D polymorphisms on selected population (10% bottom vs. 10% top population).
(0.16 MB DOC)

Table S2 Effects of the risk allelic score on traits in selected population (10% bottom vs. 10% top population).
(0.06 MB DOC)
