# Supplementary material for: Association between Type 2 Diabetes Loci and Measures of Fatness
Source: PLoS One. 2010 Jan 1;5(1):e8541. doi: 10.1371/journal.pone.0008541 (PMC2796390; doi:10.1371/journal.pone.0008541)
Supplement: Table S1 — Individual effects of T2D polymorphisms on selected population (10% bottom vs. 10% top population). (0.16 MB DOC) [file pone.0008541.s001.doc]

Table S1. Individual effects of T2D polymorphisms on selected population (10 % bottom vs. 10% top population).

| **SNP** | **Risk** | **Freq.** |  |  |  |  |  |  |  |  |  |  |  | **TRAITS** |  |  |  |  |  |  |  |  |
| --- | --- | --- | --- | --- | --- | --- | --- | --- | --- | --- | --- | --- | --- | --- | --- | --- | --- | --- | --- | --- | --- | --- |
| **Locus** | **Alle.** |  |  |  | **BMI** |  |  |  | **FMI** |  |  |  | **FAT** |  |  |  | **WC** |  |  |  | **WHR** |  |
|  |  |  |  | **β** | **se** | **p** |  | **β** | **se** | **p** |  | **β** | **se** | **p** |  | **β** | **se** | **p** |  | **β** | **se** | **p** |
|  |  |  |  |  |  |  |  |  |  |  |  |  |  |  |  |  |  |  |  |  |  |  |
| rs13266634 | C | 0.69 |  | -0.11 | 0.14 | 0.44 |  | -0.19 | 0.19 | 0.33 |  | -0.43 | 0.36 | 0.23 |  | -0.03 | 0.22 | 0.86 |  | -0.30 | 0.49 | 0.54 |
| SLC30A8 |  |  |  |  |  |  |  |  |  |  |  |  |  |  |  |  |  |  |  |  |  |  |
|  |  |  |  |  |  |  |  |  |  |  |  |  |  |  |  |  |  |  |  |  |  |  |
| rs10811661 | T | 0.87 |  | -0.16 | 0.19 | 0.39 |  | 0.06 | 0.25 | 0.81 |  | -0.64 | 0.52 | 0.23 |  | 0.16 | 0.34 | 0.65 |  | 0.13 | 0.79 | 0.86 |
| CDKN2AB |  |  |  |  |  |  |  |  |  |  |  |  |  |  |  |  |  |  |  |  |  |  |
|  |  |  |  |  |  |  |  |  |  |  |  |  |  |  |  |  |  |  |  |  |  |  |
| rs7754840 | C | 0.33 |  | 0.12 | 0.15 | 0.41 |  | -0.22 | 0.18 | 0.24 |  | -0.33 | 0.29 | 0.26 |  | 0.24 | 0.23 | 0.29 |  | -0.38 | 0.49 | 0.44 |
| CDKAL1 |  |  |  |  |  |  |  |  |  |  |  |  |  |  |  |  |  |  |  |  |  |  |
|  |  |  |  |  |  |  |  |  |  |  |  |  |  |  |  |  |  |  |  |  |  |  |
| rs4402960 | T | 0.29 |  | 0.12 | 0.14 | 0.38 |  | 0.05 | 0.19 | 0.78 |  | -0.31 | 0.34 | 0.36 |  | -0.17 | 0.24 | 0.48 |  | 0.34 | 0.48 | 0.48 |
| IGF2BP2 |  |  |  |  |  |  |  |  |  |  |  |  |  |  |  |  |  |  |  |  |  |  |
|  |  |  |  |  |  |  |  |  |  |  |  |  |  |  |  |  |  |  |  |  |  |  |
| rs1111875 | C | 0.59 |  | 0.17 | 0.14 | 0.22 |  | 0.14 | 0.19 | 0.46 |  | -.011 | 0.31 | 0.73 |  | -0.25 | 0.21 | 0.24 |  | 0.07 | 0.44 | 0.89 |
| HHEX |  |  |  |  |  |  |  |  |  |  |  |  |  |  |  |  |  |  |  |  |  |  |
|  |  |  |  |  |  |  |  |  |  |  |  |  |  |  |  |  |  |  |  |  |  |  |
| rs1801282 | C | 0.9 |  | 0.56 | 0.22 | **0.01** | ***** | 0.15 | 0.28 | 0.58 |  | 0.35 | 0.47 | 0.46 |  | 0.51 | 0.34 | 0.13 |  | 0.59 | 0.79 | 0.46 |
| PPARG |  |  |  |  |  |  |  |  |  |  |  |  |  |  |  |  |  |  |  |  |  |  |
|  |  |  |  |  |  |  |  |  |  |  |  |  |  |  |  |  |  |  |  |  |  |  |
| rs5219 | T | 0.34 |  | -0.04 | 0.14 | 0.79 |  | 0.17 | 0.19 | 0.37 |  | -0.18 | 0.31 | 0.57 |  | -0.13 | 0.22 | 0.57 |  | 0.64 | 0.44 | 0.14 |
| KCNJ11 |  |  |  |  |  |  |  |  |  |  |  |  |  |  |  |  |  |  |  |  |  |  |
|  |  |  |  |  |  |  |  |  |  |  |  |  |  |  |  |  |  |  |  |  |  |  |
| rs7903146 | T | 0.28 |  | -0.1 | 0.15 | 0.49 |  | -0.18 | 0.19 | 0.33 |  | 0.05 | 0.32 | 0.86 |  | -0.13 | 0.24 | 0.60 |  | 0.01 | 0.50 | 0.98 |
| TCF7L2 |  |  |  |  |  |  |  |  |  |  |  |  |  |  |  |  |  |  |  |  |  |  |
|  |  |  |  |  |  |  |  |  |  |  |  |  |  |  |  |  |  |  |  |  |  |  |
| rs8050136 | A | 0.43 |  | 0.40 | 0.14 | **4.3E-03** | ***** | 0.37 | 0.18 | **3.8E-02** | ***** | -0.06 | 0.30 | 0.82 |  | -0.51 | 0.43 | 0.24 |  | 0.16 | 0.23 | 0.47 |
| FTO |  |  |  |  |  |  |  |  |  |  |  |  |  |  |  |  |  |  |  |  |  |  |

All analyses are adjusted for sex and age. BMI=body mass index, FMI=fat mass index, FAT=fat %, WC=waist circumference, WHR=waist to hip ratio. β=beta coefficient, se=standard error, p= p value, *= p<0.05.
