# Supplementary material for: Association between Type 2 Diabetes Loci and Measures of Fatness
Source: PLoS One. 2010 Jan 1;5(1):e8541. doi: 10.1371/journal.pone.0008541 (PMC2796390; doi:10.1371/journal.pone.0008541)
Supplement: Table S2 — Effects of the risk allelic score on traits in selected population (10% bottom vs. 10% top population). (0.06 MB DOC) [file pone.0008541.s002.doc]

Table S2. Effects of the risk allelic score on traits in selected population (10% bottom vs.10% top population).

| **Traits** |  | **Intercept±se** | **pi** | **βs**±**se** | **ps** | **βa**±**se** | **pa** | **β±se** | **p** |
| --- | --- | --- | --- | --- | --- | --- | --- | --- | --- |
| BMI |  | -3.19±0.65 | <1.34E-06 | 0.69± 0.22 | 1.69E-03 | 0.04±0.01 | 1.19E-09 | 0.11±0.06 | 0.065 |
| FMI |  | -0.63 ±0.79 | 0.42 | -3.16±0.27 | <2E-16 | 0.03±0.01 | 4.1E-04 | 0.06±0.07 | 0.420 |
| FAT |  | 3.43±1.4 | 0.01 | -6.95±0.76 | <2E-16 | 0.01±0.01 | 0.57 | -0.16±0.12 | 0.179 |
| WC |  | -13.37±2.60 | 2.65E-07 | 8.43±1.16 | 4.33E-13 | 0.18±0.04 | 2.18E-07 | 0.09±0.18 | 0.632 |
| WHR |  | -5.40±1.01 | 9.9E-80 | 4.79±0.46 | <2E-16 | 0.09±0.01 | 1.92E-12 | -0.07±0.09 | 0.442 |

**β**s = coefficient for sex, **β**a= coefficient for age, **β**= coefficient for risk allelic score, pi= p-value for intercept, ps= p=value for sex, pa= p-value for age,

p= p-value for fatness measurement trait.
